# Kikuchi-Fujimoto Disease: A Case Report and Literature Review

**DOI:** 10.1155/2012/497604

**Published:** 2012-07-26

**Authors:** Vikrant Veer, Albert Lim, Wolfgang Issing

**Affiliations:** Otolaryngology, Freeman Hospital, NE7 7DN Newcastle upon Tyne, UK

## Abstract

*Case*. 38-year-old lady was referred to the ENT clinic with history of right-sided facial pain, otalgia, and odynophagia. Clinical examination revealed enlarged right-sided lymph nodes in the neck. Further radiological scans showed a mass near the carotid and enlarged level V lymph nodes. Lymphoma was initially suspected. Fine-needle aspiration and excision biopsy were undertaken. Histological analysis later suggested Kikuchi-Fujimoto disease, also known as histiocytic necrotising lymphadenitis. *Literature Review*. Kikuchi-Fujimoto disease (KFD) was described in 1972 as lymphadenitis with focal proliferation of reticular cells accompanied by numerous histiocytes and extensive nuclear debris. KFD, frequently found in East Asian countries, is rare in the UK. No definite aetiology of KFD is known despite autoimmune and infection factors being suggested. The diagnostic hallmark is histological findings from lymph nodes. Malignancy should be excluded. This condition is mainly self-limiting; hence, management is limited to supportive care. Steroid therapy could be used in severe cases. KFD is relatively unknown in the UK and this case report aims to highlight its occurrence in our population.

## 1. Introduction

Cervical lymphadenopathy persisting for greater than three weeks should be referred to a head and neck clinic as a suspected case of cancer according NICE guidance. In this article, we examined a case of persistent tender cervical lymphadenopathy which had confounded diagnosis until a histological specimen was obtained from the lymph tissue which encased the internal carotid artery close to the base of skull. It emerged that our patient had Kikuchi-Fujimoto disease (KFD), otherwise known as histiocytic necrotising lymphadenitis. We also performed a literature review of KFD to provide an evidence-based understanding of this disease.

## 2. Case Report

A 38-year-old Filipino nurse presented to her general practitioner in the summer of 2009 with a one-year history of right neck pain, right otalgia, and odynophagia. Clinical examination could only demonstrate a cyst on the right tonsils without any specific cervical lymphadenopathy. However, ultrasonography at that time revealed some enlarged right-sided nodes. Subsequent biopsy of the tonsillar cyst showed chronic tonsillitis with dilated crypts. A fortnight later, she presented to the otorhinolaryngology (ENT) department for uncontrolled right facial pain. A second ultrasound noted a mildly enlarged parotid gland and similar right-sided reactive cervical lymph nodes but larger in size. Head and neck radiology team proposed an MRI which then showed a 3.8 cm × 2.5 cm × 1.2 cm mass surrounding the internal carotid artery just inferior to the skull base. Other smaller lymph nodes were found at level V. Fine-needle aspirate of these level V lymph nodes found evidence of atypical cell of which low-grade lymphoma could not be excluded. Hence, an excision biopsy of these nodes was then undertaken. The diagnosis of Kikuchi-Fujimoto disease was finally suggested based on the histological findings.

## 3. Literature Review

### 3.1. History of KFD

In 1972, Dr. Masahiro Kikuchi presented a case of lymphadenitis characterised by focal proliferation of reticular cells accompanied by numerous histiocytes and extensive nuclear debris [1] in the Japanese Journal of the Haematological Society. In the same month, Dr. Fujimoto presented a similar case in a separate Japanese journal [2].

### 3.2. Epidemiology

KFD represents a condition most frequently found in East Asian and Japanese populations. The incidence is unknown but is rare in the UK and continental Europe. Extrapolating from the larger case series reports, the male to female ratio is roughly equal with slight female preponderance [3] and largely affects young adults (<30 years old).

### 3.3. Aetiology

The authors were unable to find any definitive evidence of an aetiological factor in the literature. Most articles on this subject concentrate on either an infective cause or an autoimmune disorder. 

#### 3.3.1. Infection

*Yersinia enterocolitica *[4],* Brucellosis *[5],* Bartonella henselae *[6],* Entamoeba histolytica *[7],* Mycobacterium szulgai *[8], and *Toxoplasma gondii *[9] have been isolated in case reports. However, subsequent studies have failed to support these findings. The fact that most patients with KFD are unresponsive to antibiotics suggests that these microbiological organisms were incidental findings.

Epstein-barr virus [10], herpes viruses [11–13], cytomegalovirus [14], parvovirus [15], paramyxovirus [16, 17], parainfluenza virus [17], rubella [17], hepatitis B [18] virus, Human Immunodeficiency Virus (HIV) [19], human T-lymphotropic virus type 1 [20], and the Dengue virus [21] have all been implicated in the aetiology of KFD. None of these have been found to be consistently associated with this condition [22–24].

#### 3.3.2. Autoimmune

Immunological screening of patients with KFD rarely reveals any autoimmune component in these patients. Imamura et al. in 1982 [25] performed an ultra-structural analysis of pathological tissue from patients with KFD, which drew some similarities with the histological findings in patients with systemic lupus erythematous (SLE). However, association of Kikuchi with SLE remains unclear.

There is also evidence of a genetic susceptibility for KFD. Tanaka et al. [26] found certain *human leukocyte antigen* (HLA) class II genes were more common found in those with KFD. These genes were found to be more common in Asian populations compared to Caucasian groups. The implication of this evidence is that KFD represents a self-limiting autoimmune response to an upper respiratory tract viral infection in genetically-susceptible individuals. Some evidence suggests that KFD can initiate the onset of SLE, or is a short-lived version of SLE [27–33].

## 4. Clinical Features

The typical pattern of presentation is one of general flu-like symptoms with posterior cervical, tender lymphadenopathy. Other less common nonspecific symptoms are headache, nausea, fatigue, and arthralgia. The disease process continues for approximately two to three months before resolving spontaneously [34–36].

## 5. Diagnosis

The diagnosis of KFD is rather problematical due to its relative obscurity, nonspecific symptoms, and imprecise histological diagnosis [37]. Currently, the only reliable method of diagnosis is histological examination of a lymph node excision biopsy. Unfortunately, samples from a fine-needle aspiration are generally not sensitive enough to provide a reliable diagnosis with an overall accuracy of 56.3% [38]. Blood tests classically show a mild neutropenia with mildly raised in the C-reactive protein (CRP) and erythrocyte sedimentation rate (ESR) [37]. 

**Figure 1:**
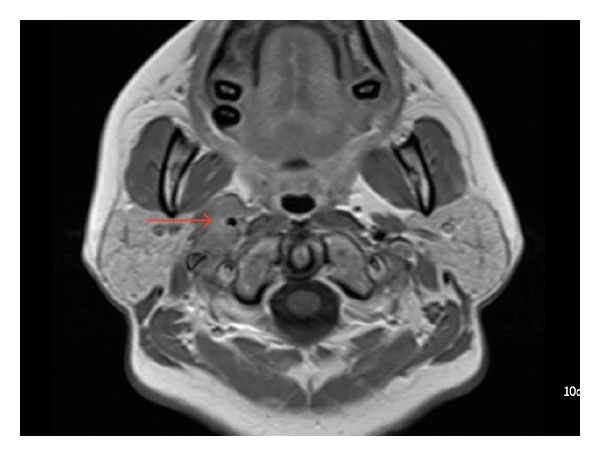
MRI scans of our patient showed multiple right-sided swollen lymph nodes. They were subsequently biopsied.

Imaging techniques such as CT, MRI, PET/CT scanning also rarely provide conclusive diagnostic results [39–42]. An incorrect provisional diagnosis of lymphoma or tuberculosis is made in many instances. In a study of 96 retrospective scans of confirmed KFD, Kwon et al. [43] found that on CT scanning cases of KFD had the following typical appearances:

- multiple homogeneous lymphadenopathy involving levels II to V,
- 94% were smaller than 2.5 cm, this allows some differentiation from lymphoma which typically produces few but larger nodes,
- perinodal infiltration and necrosis is commonly found (Figure 1).

Ultrasonography scans frequently show lymph nodes with a hypoechoic center and a hyperechoic rim [44]. Again, these features have a low specificity for KFD [37, 44]. These findings certainly do not allow the diagnosis of KFD with imaging techniques. However, linked to the clinical history, one may at least be suspicious of KFD rather than a neoplastic lesion. 

### 5.1. Histological Findings

A 2010 study by Kim et al. noted the following in cutaneous Kikuchi disease patients:

- slight predominance of CD8^+^ lymphocytes,
- lymphohistiocytic infiltration and nonneutrophilic karyorrhexis (usually).

**Figure 2:**
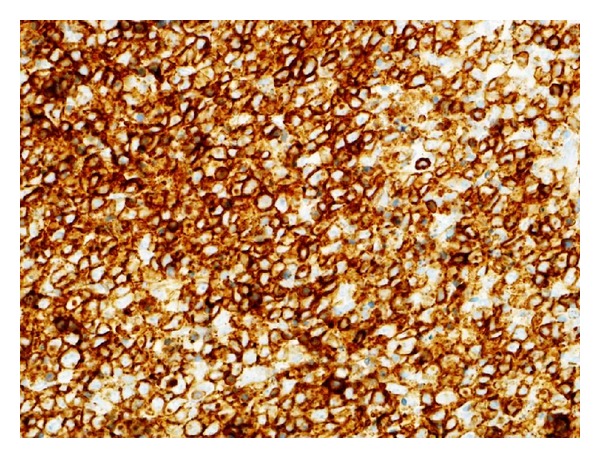
Histological staining for CD8+ lymphocytes infiltration of right-sided swollen lymph node biopsy.

The immunophenotype of Kikuchi disease is primarily composed of mature CD8-positive and CD4-positive T lymphocytes. Lymphocytes and histiocytes also exhibit a high rate of apoptosis. There are relatively few B cells and natural killer (NK) cells are present. Positive immunostaining results by monoclonal antibody Ki-M1P are seen in Kikuchi disease but not in malignant lymphoma (Figure 2).

### 5.2. Differential Diagnoses

These are among

- lymphoma,
- systemic lupus erythematous (SLE),
- infectious mononucleosis,
- kawasaki disease,
- sarcoidosis,
- tuberculosis,
- syphilis.

## 6. Management

KFD is a self-limiting condition that rarely requires specific treatment in most cases. Management is, therefore, based on supportive therapy, such as analgesia and anti-inflammatory medication. In patients with neurological symptoms or in those cases where KFD is found with another medical condition, immunosuppression with corticosteroids appears to improve the patient's condition rapidly. A high initial oral dose of prednisolone with a subsequent reducing dose is the advocated regime [45, 46]. There are reports of excellent responses to hydroxychloroquine [47, 48], immunoglobulins [49], and minocycline [50]. Recurrence of KFD is approximately 3% [3].

## 7. Summary

KFD is a rare condition, particularly in the Western world. There is a substantial amount of research into KFD. Unfortunately, the aetiology, pathology, diagnosis, and management of this disease are all still relatively uncertain and are subject to debate. Further scientific studies in this disease may also provide valuable insight into a number of other autoimmune conditions.
